# Psyllium Supplementation in Adolescents Improves Fat Distribution & Lipid Profile: A Randomized, Participant-Blinded, Placebo-Controlled, Crossover Trial

**DOI:** 10.1371/journal.pone.0041735

**Published:** 2012-07-27

**Authors:** Martin de Bock, José G. B. Derraik, Christine M. Brennan, Janene B. Biggs, Greg C. Smith, David Cameron-Smith, Clare R. Wall, Wayne S. Cutfield

**Affiliations:** 1 Liggins Institute, University of Auckland, Auckland, New Zealand; 2 Department of Molecular Genetics, University of Auckland, Auckland, New Zealand; 3 Department of Nutrition, University of Auckland, Auckland, New Zealand; National Institute of Agronomic Research, France

## Abstract

**Aims:**

We aimed to assess the effects of psyllium supplementation on insulin sensitivity and other parameters of the metabolic syndrome in an at risk adolescent population.

**Methods:**

This study encompassed a participant-blinded, randomized, placebo-controlled, crossover trial. Subjects were 47 healthy adolescent males aged 15–16 years, recruited from secondary schools in lower socio-economic areas with high rates of obesity. Participants received 6 g/day of psyllium or placebo for 6 weeks, with a two-week washout before crossing over. Fasting lipid profiles, ambulatory blood pressure, auxological data, body composition, activity levels, and three-day food records were collected at baseline and after each 6-week intervention. Insulin sensitivity was measured by the Matsuda method using glucose and insulin values from an oral glucose tolerance test.

**Results:**

45 subjects completed the study, and compliance was very high: 87% of participants took >80% of prescribed capsules. At baseline, 44% of subjects were overweight or obese. 28% had decreased insulin sensitivity, but none had impaired glucose tolerance. Fibre supplementation led to a 4% reduction in android fat to gynoid fat ratio (p = 0.019), as well as a 0.12 mmol/l (6%) reduction in LDL cholesterol (p = 0.042). No associated adverse events were recorded.

**Conclusions:**

Dietary supplementation with 6 g/day of psyllium over 6 weeks improves fat distribution and lipid profile (parameters of the metabolic syndrome) in an at risk population of adolescent males.

**Trial Registration:**

Australian New Zealand Clinical Trials Registry ACTRN12609000888268

## Introduction

The metabolic syndrome encompasses a set of biochemical and physical parameters that are associated with a greater risk for the development of type 2 diabetes mellitus and cardiovascular disease, and all cause mortality [1]. These parameters include increased central adiposity, and adverse changes in blood pressure, lipid profile, and insulin sensitivity [2]. The emergence of the metabolic syndrome in the paediatric population is primarily a result of dramatic increases in childhood obesity [3], and tracks from adolescence into adulthood [4]. Therefore, a range of initiatives are frequently employed in the attempt to decrease the incidence of the metabolic syndrome and obesity in childhood. These are mostly community-based interventions, aiming to foster increased physical activity and dietary changes. Nutritional management in particular, varies greatly (from caloric restriction to changes in macronutrient composition and energy ratio), as there is a lack of consensus on the optimal approach [5].

In children, the effects of dietary fibres on parameters of the metabolic syndrome are not well established. Cross sectional data have shown that fibre and whole grain consumption in adolescence is associated with increased insulin sensitivity [6] and a lower incidence of the metabolic syndrome [7]. However, the majority of clinical studies have focused on dietary fibre combined with either exercise and/or other dietary interventions [8], [9]. Thus, to our knowledge, there have been no placebo-controlled clinical trials investigating the effect of supplementation with dietary fibre alone on parameters of the metabolic syndrome in adolescents.

Dietary fibres encompass a broad array of compounds (primarily of plant origin) with known physiological benefits, including laxation, and improvements in glucose homeostasis and cholesterol [10]. The gel-forming water-soluble fibres are those that appear to have the most beneficial effects on post-prandial glycemia [11]. Such fibres include the seed husks of psyllium (*Plantago* spp., in particular *P. ovata*), also known as ispaghula, which is often used to enrich cereals and other food items. Psyllium husks encompass a mixture of neutral and acid polysaccharides containing galacturonic acid, with a 70/30 ratio of soluble/insoluble fibre. Psyllium has been used safely in children and adolescents, and was shown to improve hypercholesterolemia [12], [13], [14]. In this study, we aimed to investigate the effect of psyllium fibre supplementation alone on insulin sensitivity and other parameters of the metabolic syndrome in an at risk adolescent population.

## Materials and Methods

### Ethics Approval

Ethics approval for this study was provided by the Northern Y Regional Ethics Committee. Written informed consent was obtained from participants and caregivers.

### Subjects

**Figure 1:**
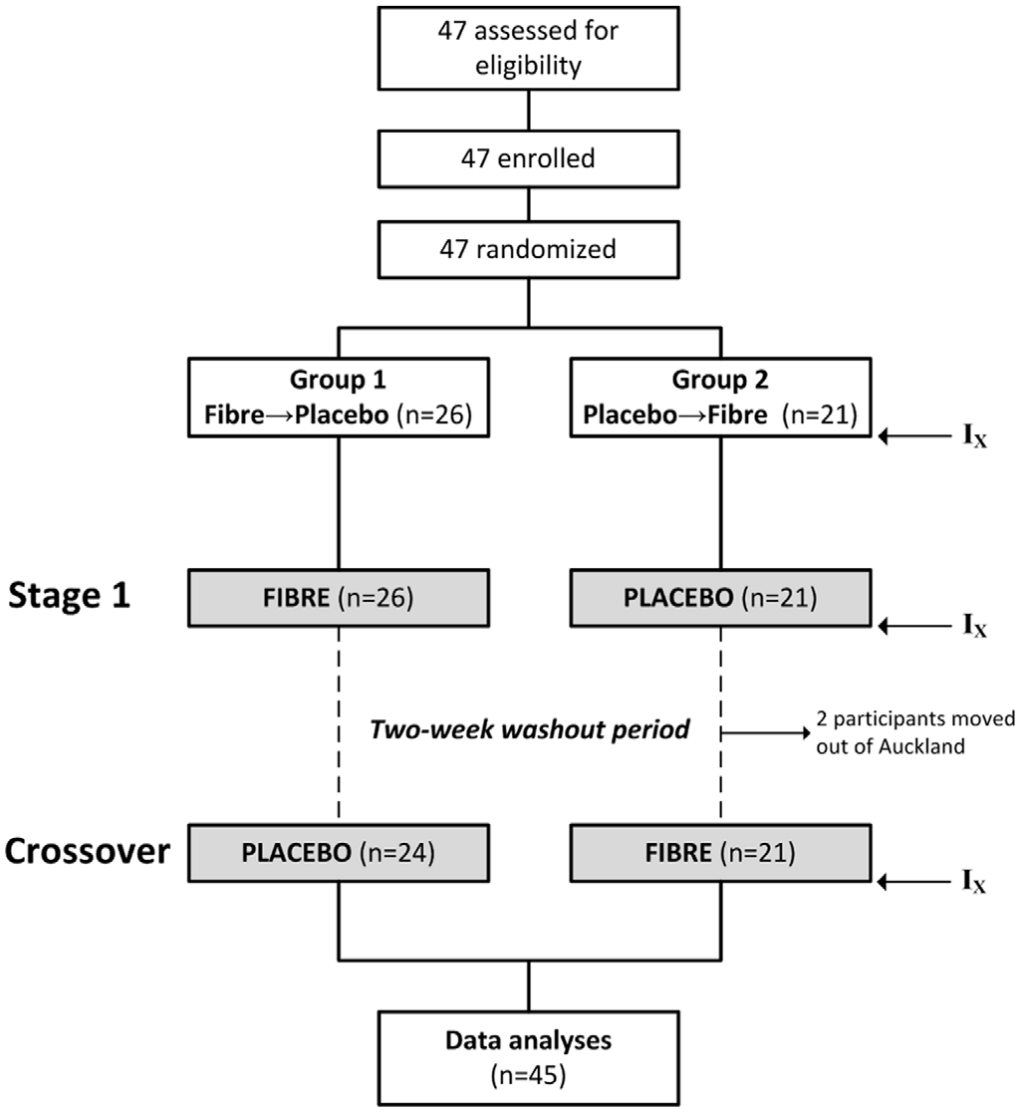
Summary of study’s recruitment process and trial execution. I_X_ indicates timing of assessments.

Healthy adolescent males aged 15–16 years were recruited from high schools in Auckland (New Zealand) to participate in the study (Figure 1). We targeted schools in lower socio-economic areas with high rates of obesity, in order to select adolescents at greater risk of developing the metabolic syndrome. Exclusion criteria included those receiving medications that alter glucose metabolism (e.g. steroids, stimulants, and insulin), and smokers. Participants provided written informed consent if they were 16 years and over.

### Study Design

This study was a randomized, participant-blinded, placebo-controlled, crossover trial. The protocol for this trial and supporting CONSORT checklist are available as supporting information (see Checklist S1 and Protocol S1).

Randomization and allocation to trial group were done using computer random number generation. All participants were randomized into a 6-week intervention with either 6 g/day of psyllium (*P. ovata*) (equating to 6 g of dietary fibre) or 6 g/day of potato starch placebo (Figure 1). The dose of 6 g/day was adopted based on review of the existing literature [15], as well as on the volume of fibre and placebo each dose would equate to, so as not to affect compliance with study protocol. After a 2-week washout period, participants crossed over to receive the opposite intervention for a further six weeks (Figure 1). Both the psyllium and potato starch were packed as 500 mg capsules (Douglas Pharmaceuticals, Auckland, New Zealand). The capsules were blister-packed to aid compliance, and participants were instructed to consume the 12 capsules per day with large amounts of water. Capsules could be consumed all at once or divided in doses, and with or without food. Adherence to dosing was monitored by counting non-consumed capsules in returned blister packs at the end of each 6-week intervention. Participants were advised to continue their normal eating and exercise patterns during the study period.

### Study Parameters

All clinical assessments were carried out at the Maurice & Agnes Paykel Clinical Research Unit (Liggins Institute, University of Auckland). Subjects were assessed at three time points after an overnight fast: baseline, end of the first 6-week intervention, and end of the second 6-week intervention (Figure 1). Height was measured using a Harpenden stadiometer. Weight and body composition were assessed using both body mass index (BMI) and whole-body dual-energy x-ray absorptiometry (Lunar Prodigy 2000, General Electric, Madison, WI, USA). Body composition data of interest were total percentage body fat and the ratio of android fat to gynoid fat. Note that android and gynoid fat values were determined by the manufacturer’s software, based on an automated sectioning of specific areas of the body [16]. BMI data were converted to standard deviation scores (BMI SDS) according to British 1990 standards [17].

After an overnight fast, blood samples were obtained to assess metabolic factors. Glucose, triglycerides, cholesterol, HDL, and LDL concentrations were measured on a Hitachi 902 autoanalyser (Hitachi High Technologies Corporation, Tokyo, Japan) by enzymatic colorimetric assay (Roche, Mannheim, Germany) with an interassay CV of less than 2.5%. Insulin concentrations were measured using an Abbott AxSYM system (Abbott Laboratories, Abbott Park, IL, USA) by microparticle enzyme immunoassay with an interassay CV of 5.4%. Insulin sensitivity was assessed by a 75 g oral glucose tolerance test using the Matsuda method, with glucose and insulin samples collected at 0, 30, 60, 90, and 120 minutes [18]. The Matsuda method has a strong correlation with the hyperinsulinemic euglycaemic clamp (r = 0.77) [19], and excellent reproducibility during multiple measures [20].

24-hour ambulatory blood pressure was assessed prior to each clinical assessment. Participants were fitted with a Spacelabs 90207 or 90217 (Spacelabs Medical Inc., Redmond, USA), with each subject being assigned the same device model for all assessments. Measurements were performed every 20 minutes from 0700–2200, and every 30 minutes from 2200–0700. Only profiles with a total of at least 40 readings over a 24-hr period were included for analysis [21].

Three dietary records were collected at baseline and at clinical assessment following each 6-week intervention. Each dietary report encompassed an itemized nutritional intake recorded during two school days and one weekend day. Nutritional intake was recorded using standard household measures, as well as the information from food labels where appropriate. Participants were instructed by a nutritionist [CRW] on how to fill out the food diary accurately. A trained investigator [MdB] reviewed all food records with each participant to address unclear descriptions, errors, omissions, or doubtful entries. Records were subsequently entered into Foodworks software (v6.0, Xyris Software, Brisbane, Australia) by the trained investigator [MdB]. Accuracy of food record entry was also externally confirmed by the nutritionist [CRW], randomly selecting and verifying 10% of all records.

Physical activity was assessed using the Physical Activity Questionnaire for Adolescents (PAQ-A) (University of Saskatchewan, Saskatoon, Canada). Leisure activities were modified to reflect those engaged by New Zealand youth. This self-administered 7-day recall questionnaire has been validated for use in adolescents [22].

Demographic data were also collected on all subjects. Socio-economic status (SES) was classified using the New Zealand Index of Deprivation 2006 (NZDep2006) [23]. This uses household census data reflecting nine aspects of material and social deprivation to divide New Zealand into tenths (scored 1–10) by residential address, where a higher score reflects lower SES [23].

### Statistical Analysis

Baseline data associations were assessed using simple linear regressions, but the association between SES and insulin sensitivity was examined using non-parametric Spearman’s rank correlation. Baseline analyses were carried out in Minitab v.16 (Pennsylvania State University, State College, PA, USA). Crossover trial data were analysed in SAS v.9.2 (SAS Institute, Cary, NC, USA) using a linear mixed model design based on repeated measures, which accounted for treatment sequence (Placebo→Fibre vs Fibre→Placebo), treatment phase (Stage 1 vs Crossover; Figure 1), ethnicity, SES, as well as participant as a random factor. Importantly, models also incorporated the baseline value of the outcome response as a co-variate, to account for the different starting points for each subject at the beginning of the study. The Johnson transformation was adopted when necessary to stabilize the variance. Data are expressed as means ± SEM.

## Results

A total of 47 healthy adolescent males (aged 15.8±0.1 years at baseline) met the inclusion criteria and were enrolled in the study. Randomization order was established prior to recruitment of subjects, and we aimed for a minimum of 42 subjects (i.e. 21 in each group) as required by the power calculation to detect a 25% change in insulin sensitivity [24]. At the point at which we had enrolled and successfully studied 45 subjects it was obvious that study failure rates were far lower than anticipated, thus recruitment was stopped. This explains the uneven ratio of subjects randomly allocated between groups 1 and 2 (Figure 1). Subsequently, two participants were lost to follow up, and were excluded (Figure 1).

All participants were from areas of relatively low SES, with 44% from the lowest quintile of SES in New Zealand. Subjects were of Pacific Island (46%), European (37%), Maori (15%), and Indian (2%) ethnicities. Mean BMI at baseline was 25.8±0.7 kg/m^2^, with 24% of subjects obese (BMI ≥30 kg/m^2^) and a further 20% overweight (BMI ≥25 but <30 kg/m^2^); mean percentage body fat was 23.5±1.7%. Participants’ compliance with the study was very high: 87% of participants took more than 80% of prescribed capsules over the 12 weeks of intervention. No associated adverse events (including gastrointestinal) were recorded during this study.

Mean pre-study dietary fibre intake was 23.1±1.7 g/day (Table 1), with only 37% of subjects meeting the recommended daily intake of 28 g/day for this age group [25]. As a result, the addition of 6 g/day of psyllium during the treatment period equated to a mean individual increase in daily dietary fibre intake of 36.4±4.6%, with an equivalent 50% or more increase recorded in four subjects. Baseline data demonstrate a high intake of energy derived from fat, including saturated fat (Table 1).

**Table 1:** Baseline daily dietary parameters among study subjects.

Dietary parameter	Mean ± SEM
**Total energy (kJ)**	10673±560
**Fibre (g)**	23.1±1.7
**Energy from fat (%)**	35.9±1.2
**Energy from saturated fat (%)**	15.9±0.7
**Energy from carbohydrates (%)**	43.5±1.6
**Energy from sugars (%)**	16.2±1.1
**Energy from protein (%)**	17.0±1.2

### Baseline

**Figure 2:**
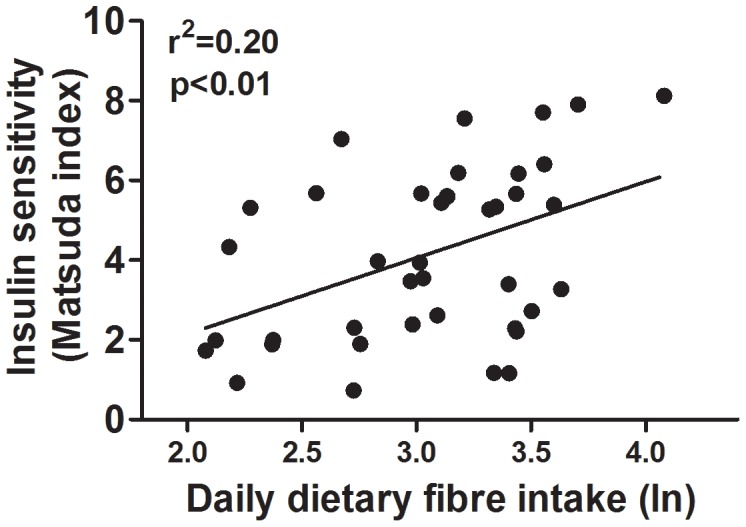
The association between baseline daily dietary fibre intake (log-transformed) and insulin sensitivity (Matsuda index).

**Figure 3:**
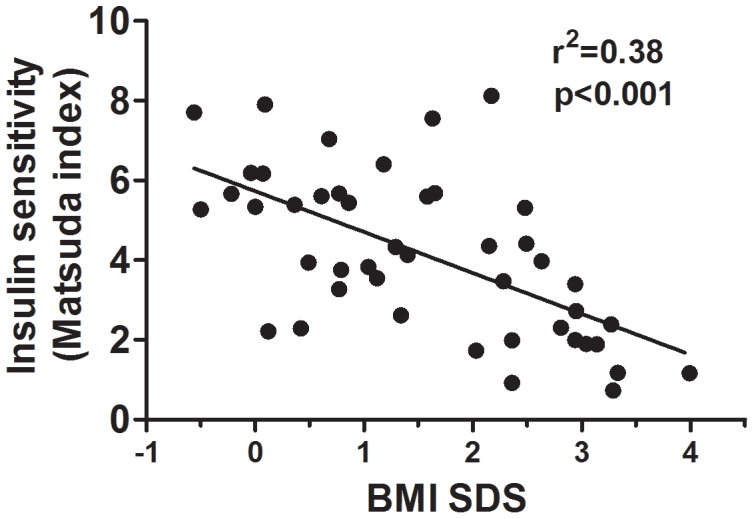
The association between BMI SDS and insulin sensitivity (Matsuda index) at baseline.

Insulin sensitivity at baseline was positively associated with mean daily intake of dietary fibre (r^2^ = 0.20; p<0.01; Fig. 2), and inversely associated with BMI SDS (r^2^ = 0.38; p<0.001; Fig. 3). SES was also correlated with insulin sensitivity (p = 0.037), so that the higher the index of deprivation the lower the Matsuda index (ρ = −0.31). 28% of subjects were insulin resistant with a baseline Matsuda score lower than 2.5. BMI SDS was associated with baseline triglycerides (r^2^ = 0.24; p<0.001), total cholesterol (r^2^ = 0.26; p<0.001), LDL (r^2^ = 0.26; p<0.001), HDL:LDL ratio (r^2^ = 0.19; p<0.01), but not HDL (r^2^ = 0.00; p = 0.65) concentrations.

### Crossover Trial

Dietary intake among individual participants did not change significantly throughout the study. Thus, total caloric intake (p = 0.43), total fibre intake (p = 0.44), and the percentage of total calories from saturated fat (p = 0.17) at baseline were not different to the respective intake consumed during placebo and fibre treatment. In addition, there was also no change in physical activity levels among groups throughout the study.

Although fibre supplementation did not lead to a reduction in weight, BMI SDS, or body fat percentage, it did lead to a 4% reduction in android fat to gynoid fat ratio (p = 0.019; Table 2). Psyllium supplementation also led to a 0.12 mmol/l (6%) reduction in LDL cholesterol (p = 0.042; Table 2). There were no observed effects on insulin sensitivity, fasting plasma insulin, or glycemic status (i.e. fasting plasma glucose), irrespective of ethnicity, baseline fibre intake, or BMI SDS. Ambulatory blood pressure parameters were similar with placebo and fibre intake, except nighttime systolic blood pressure that tended to be on average 3.1 mmHg lower with psyllium supplementation (p = 0.073; Table 2).

**Table 2:** Outcome measures following a 6-week supplementation with 6 g/day of psyllium fibre or placebo.

Variable	Placebo	Fibre	p-value
**Anthropometry**			
Weight (kg)	83.4±3.1	83.0±3.0	0.65
BMI (kg/m^2^)	26.2±1.0	26.0±0.9	0.92
% body fat	23.8±1.7	23.4±1.7	0.95
Android fat to gynoid fat ratio	0.99±0.04	0.95±0.04	0.019
**Ambulatory blood pressure**			
Daytime diastolic (mmHg)	69.8±0.9	69.8±0.9	0.86
Daytime systolic (mmHg)	123.9±1.5	122.6±1.3	0.44
Nighttime diastolic (mmHg)	56.2±1.1	55.1±0.8	0.57
Nighttime systolic (mmHg)	109.4±1.7	106.3±1.3	0.073
Diastolic dip (%)	19.2±1.4	20.3±1.4	0.58
Systolic dip (%)	11.4±1.2	13.6±1.0	0.13
**Plasma**			
Glucose (mmol/l)	5.20±0.07	5.11±0.06	0.19
LDL (mmol/l)	2.46±0.09	2.32±0.09	0.042
HDL (mmol/l)	1.19±0.04	1.17±0.04	0.40
HDL to LDL ratio	0.53±0.03	0.54±0.03	0.59
Triglycerides (mmol/l)	0.95±0.06	0.94±0.06	0.99
**Hormones**			
Insulin (µU/l)	14.8±1.5	15.3±1.5	0.51
**Insulin sensitivity** (Matsuda index)	3.88±0.3	3.85±0.3	0.90

Data are means ± SEM, and p-values refer to results from multivariate models.

## Discussion

This is the first randomized, participant-blinded, placebo-controlled, crossover trial investigating the effects of psyllium supplementation on parameters of the metabolic syndrome in adolescents. Our data show that even in the context of a relatively short intervention, psyllium supplementation improves LDL cholesterol and android fat to gynoid fat ratio. Conversely, there was no improvement in insulin sensitivity and HDL, which are other parameters of the metabolic syndrome. These results have public health implications as commercial food manufacturers often use psyllium to fortify products such as cereal and baked goods to boost their fibre content.

Our study corroborates previous data showing that psyllium has lipid lowering properties in children and adolescents. The 6% improvement in LDL cholesterol concentrations we observed is comparable to other studies that have shown improvements of 0–23% using psyllium doses ranging from 5–25 g/day [12], [13], [14], [26]. The lipid lowering action of soluble fibres such as psyllium occurs by binding bile acids and cholesterol, increasing faecal excretion of bile salts, and reducing cholesterol synthesis via production of short-chain fatty acids [27]. Importantly for this study, the reduction of LDL provides evidence that psyllium can be absorbed in the more palatable capsulated form.

We also observed a reduction in the android to gynoid ratio of fat distribution with fibre supplementation, which indicates a decrease in central adiposity. Similarly, a recent large descriptive study in adolescents showed decreased visceral fat among subjects with the highest fibre intake [28]. Thus, although we observed no change in BMI SDS, our findings are important as central obesity is an independent risk factor for the development of the metabolic syndrome and associated cardiovascular disease risk [29]. Possible explanations for the observed effect in fat distribution include altered dietary fat lipolysis and subsequent absorption [30], or modulation of sex steroids that effect fat distribution [31]. Importantly, the results could not be explained by changes in exercise patterns.

In this study, psyllium supplementation over 6 weeks did not affect insulin sensitivity. However, previous studies in adults with type 2 diabetes showed that food supplementation with psyllium led to improved glucose metabolism, as examined by post-prandial glucose and insulin excursion [32], [33]. This improvement is likely explained by the solubility and viscosity of psyllium, which sequesters carbohydrate absorption [11], and delays gastric emptying and intestinal transit time [34]. In contrast, our study investigated the effects of psyllium on insulin sensitivity in the longer term. Anderson et al. have previously shown that supplementation with 10.2 g/day of psyllium over three days improves post-prandial glucose concentrations, but not insulin sensitivity (measured by euglycaemic hyperinsulinaemic clamp) in adults with type 2 diabetes [33]. Changes in insulin sensitivity would require additional physiological properties of psyllium, such as the production of short-chain fatty acids [35]. Thus, our null result may be explained by the poor fermentation of psyllium to produce short-chain fatty acids as compared to other sources of dietary fibre [36], [37]. However, the effects of short-chain fatty acids on insulin sensitivity are questionable [38], and these may even be deleterious in the long-term as observed in animal models [39]. A further possible explanation (and a weakness of our study) relates to our chosen method to assess insulin sensitivity; i.e. we adopted an oral glucose tolerance test rather than the labour-intensive gold standard euglycaemic hyperinsulinaemic clamp. One trial examining the effect of resistant starch on insulin sensitivity detected an improvement using the clamp technique, but did not demonstrate a difference using the Homeostasis Model Assessment (HOMA) proxy [40].

Commercial food producers have capitalised on the broad benefits of fibre, commonly using psyllium to enrich cereals and other foods. By definition, fibre encompasses a broad range of edible plant compounds, which have physiological health benefits including laxation, lowered cholesterol, and improved glucose metabolism [10]. However, given that dietary fibre encompasses such a diverse range of compounds, there is a wide variation in their physiological effects. The implication is that while psyllium is a highly soluble and palatable fibre that can easily be added to food products, it may not deliver all the health benefits associated with the consumption of different forms of fibre. While we do not dispute the overall benefits of dietary fibre, it is important that consumers and food producers become aware that not all forms of fibre are equal in terms of physiological action.

The adequate intake for dietary fibre for adolescents is 28 g/day in Australia-New Zealand [25]. Dietary fibre intake in our study population was poor, as only 37% of participants consumed 28 g/day or more. Our observation is not unusual, and similar figures have been obtained for other adolescent populations [41]. These findings are reason for concern, as a recent cross-sectional study in adolescents showed that those in the highest quintile of fibre intake had a three-fold reduction in the incidence of the metabolic syndrome compared to those in the lowest quintile [7].

In conclusion, we showed that fibre supplementation using psyllium improves fat distribution and lipid profile, even after a relatively short intervention of six weeks. Conversely, psyllium supplementation did not improve insulin sensitivity. Due to the enormous burden that cardiovascular diseases have on public health, our findings have potentially important public health implications. Continued awareness and promotion of the value of dietary fibre in the adolescent diet is required. It is possible that commercial food manufacturers, through fortification of food with dietary fibre such as psyllium, could play a role in the prevention of cardiovascular diseases. However, further research is warranted to investigate the best types of fibre, delivery method, dose, and length of treatment to determine the appropriate fibre supplementation and associated health benefits.

## Supporting Information

Protocol S1 **Trial Protocol.**
(PDF)

Checklist S1 **CONSORT Checklist.**
(PDF)
