# Supplementary material for: Psyllium Supplementation in Adolescents Improves Fat Distribution & Lipid Profile: A Randomized, Participant-Blinded, Placebo-Controlled, Crossover Trial
Source: PLoS One. 2012 Jul 27;7(7):e41735. doi: 10.1371/journal.pone.0041735 (PMC3407232; doi:10.1371/journal.pone.0041735)
Supplement: Protocol S1 — Trial Protocol. (PDF) [file pone.0041735.s001.pdf]

NATIONAL APPLICATION FORM FOR ETHICAL APPROVAL OF A RESEARCH PROJECT

Ethics reference number and date received
(for office use only)

Part 1: Basic Information

1. Full project title (include protocol number if applicable)

Can supplemented dietary fibre improve insulin sensitivity in obese adolescents?

A randomized cross-over trial of a high fibre isocaloric diet compared to an isocaloric diet alone in an adolescent community with a high incidence of obesity

2. Short project title (lay title)

Can a high fibre diet reduce the amount of insulin the body needs to process the sugar consumed in meals?

3. Principal investigator's name and position

Dr Martin Isaac de Bock, Endocrinology Fellow, Liggins Institute.

4. Contact address of principal investigator

Address		
3A/10 Laxon Terrace	Work phone no.	021 1826579
Newmarket	Emergency no.*	021 1826579
Auckland	Fax	
1050	Email	Martin-d@clear.net.nz

5. Principal investigator's qualifications and experience in the past five years (relevant to proposed research)

Qualifications: MBCHB, university of Auckland. Diploma of Paediatrics, university of Auckland.

Experience: Clinical duty in Paediatric medicine at Taranaki Base Hospital, Starship Hospital, National Womens Hospital, Middlemore Hospital. This has included 4 months exclusive pediatric endocrinology, where 60% of the workload is diabetes related. General paediatric work at Taranaki, Middlemore, and Starship has included work with obese chidren and management strategies for weight reduction.

6. Co-investigator's name(s), qualifications and position(s) and,. if more than one locality; principal investigator at **each** locality

A	Professor Wayne Cutfield, MBChB, DCH, FRACP, director of Liggins Institute
B	Dr Paul Hofman, MBChB, FRACP, Associate Professor in Paediatrics. Paediatric Endocrinologist
C	Dr Craig Jefferies MBCHB, FRACP, Paediatrics Senior Lecturer. Paediatric Endocrinologist.
D	
E	
F	
G	

7.1 Address of A above

The Liggins Institute, The University of Auckland, Private Bag 92019, Auckland 1142, New Zealand	Work phone no.	3737599 , ext 86691
	Emergency no.*	021734441
	Fax	3738763
	Email	w.cutfield@auckland.ac.nz

7.2 Address of B above

The Liggins Institute, The University of Auckland, Private Bag 92019, Auckland 1142, New Zealand	Work phone no.	3737599
	Emergency no.*	021938897
	Fax	3738763
	Email	P.hofman@auckland.ac.nz

7.3 Address of C above

The Liggins Institute, The University of Auckland, Private Bag 92019, Auckland 1142, New Zealand	Work phone no.	3737599
	Emergency no.*	021 938827
	Fax	3738763
	Email	CraigJ@adhb.govt.nz

7.4 Address of D above

	Work phone no.	
	Emergency no.*	
	Fax	
	Email	

7.5 Address of E above

	Work phone no.	
	Emergency no.*	
	Fax	
	Email	

7.6 Address of F above

	Work phone no.	
	Emergency no.*	
	Fax	
	Email	

7.7 Address of G above

	Work phone no.	
	Emergency no.*	
	Fax	
	Email	

(* option for ethics committee's information only)

8. Where this is supervised work

8.1 Supervisor's name	Professor Wayne Cutfield, MBChB, DCH, FRACP,
Position	director of Liggins Institute
Daytime phone number	3737599 , ext 86691

8.2 Signature of supervisor (where relevant)<br>Declaration: I take responsibility for all ethical aspects of the project	

9. List locality organisation/s involved, including contact address, and complete the locality assessment in Part 4: Declarations (refer to the Guidelines (NAFG-2009-v1))

Research insititue:

The Liggins Institute, The University of Auckland, Private Bag 92019, Auckland 1142, New Zealand

Recruitment from:

Decile 1-3 high schools affiliated with the Liggins Institute Educational Network of Science (LENScience). Contact person – Jackie Bay.

10. I wish the protocol to be heard in a closed meeting. [ ] Yes [X] No

If the answer is yes, please provide a reason why you wish the protocol to be heard in a closed meeting in accordance with the Official Information Act 1982.

11. If the study is based, in part or in full, overseas, which countries are involved?

No

12. Has this application been reviewed by another ethics committee in New Zealand or overseas? [ ] Yes [X] No

(If yes, advise which country, the name of the committee/s and the decision/s of the committee/s)

Please note a copy of the report/s may be requested.

13. **Human tissue** – Does the project involve collection or use of human tissue? If **yes**, complete Part 5. [X] Yes [ ] No

14. **Gene studies** – Does this research involve any gene or genetic studies? If **yes**, complete Part 6. [ ] Yes [X] No

15. **Xenotransplantation** – Does this research involve the transplantation of living biological material from one species to another? [ ] Yes [X] No

If **yes**, complete Part 7.

16. **Consent** – Are all participants able to provide consent for themselves? If **no**, complete Part 8. [ ] Yes [X] No

17. **Lay summary** – give a brief **lay (non-technical)** summary of the study (not more than 200 words) such as you would give as an explanation to participants.

45 people will be recruited to the study from decile 1-3 high schools affiliated with the Liggins Institute Educational Network of Science (LENScience). Each participant will be randomly assigned to one of two groups. One of the groups will have 6 grams per day of fibre added to their diet, while the other group eats as normal. The fibre will be taken dissolved as a drink. After 6 weeks the two groups will cross over. Measurements of height and weight, blood pressure, pulse, and a blood test measuring insulin sensitivity will occur before the study, at cross over, and at the end. All participants will be given standard advice about exercise and healthy eating if they are overweight. Participants will also be asked to recall what they ate for 24 hour period at the three times they are assessed. At the end of the study we will look at the data to see if the amount of insulin needed by the body to process sugar is affected by the extra fibre in the diet.

18. Proposed starting date (dd/mm/yy): January 2010

19. Proposed finishing date (dd/mm/yy): December 2010

20. Duration of project in New Zealand (mm/yy): 12 months

21. Proposed final report date (mm/yy): 12/10

22. Has the clinical trial been registered? [X] Yes [ ] No

If **yes**, name the register: Australian and New Zealand Clinical Trial Registry

If **no**, has registration been applied for? [ ] Yes [ ] No

Comment:

Part 2: Ethical Principles

A. Validity of research *(Operational standard paragraphs 53–59)*

SCIENTIFIC BASIS

A1. Aims of the project

A1.1 What is the hypothesis/research question(s) and/or the specific aims of the project? (State briefly.)

Comment:
Research question:
Can supplemented dietary fibre improve insulin sensitivity in obese adolescents?
Aims:
1. To investigate the effect of a high fibre diet on insulin sensitivity (S_I) in an adolescent population with a high incidence of obesity.
2. To determine whether a high fibre diet improves S_I independently of weight loss.
3.To examine the effects of a high fibre diet on body composition, arterial distensibility, lipids and 24-hour ambulatory blood pressure.

Key articles for reference:

1. Pereira M, Jacobs DJ, Pins J, Raatz S, Gross M, Slavin J, et al. Effect of whole grains on insulin sensitivity in overweight hyperinsulinemic adults. The American Journal of Clinical Nutrition. 2002;75(5):848 – 55. *(shows improved insulin sensitivity in adults with high fibre diet, the effect being greater in the more obese)*
2. Saely C, Aczel S, Marte T, Langer P, Hoefle G, Drexel H. The metabolic syndrome, insulin resistance, and cardiovascular risk in diabetic and nondiabetic patients. The Journal of Clinical Endocrinology and Metabolism. 2005;90(10):5698 - 703. *(highlights the importance of insulin resistance being an independent (from obesity) risk factor for cardiovascular disease)*
3. Thomas D, Elliot E, Baur L. Low glycaemic index of low glycaemic load diets for overweight and obesity. Cochrane Database of Systematic Reviews. 2007(3):CD005105. *(in diets with low glycaemic index or glycaemic load found that the low-carbohydrate diet reduced body mass, body mass index (BMI) and total cholesterol more than conventional low-fat diets. Also highlights the lack on consensus on diet intervention for obesity management)*

4. Steinberger J, Moran A, Hong C, Jacobs DJ, Sinaiko A. Adiposity in childhood predicts obesity and insulin resistance in young adulthood. Journal of Pediatrics. 2001;138(4):469 - 73.
5. Arslanian S, Suprasongsin C. Insulin sensitivity, lipids, and body composition in childhood: is "syndrome X" present? The Journal of Clinical Endocrinology and Metabolism. 1996;81(3):1058 - 62.
6. Caprio S, Bronson M, Sherwin R, Rife F, Tamborlane W. Co-existence of severe insulin resistance and hyperinsulinaemia in pre-adolescent obese children. Diabetologia. 1996;39(12):1489 – 97

These three articles link obesity with insulin resistance in children and adolescence – highlighting the requirement to investigate ways to improve insulin resistance in obese in children and adolescents.

7. Slavin J, Martini M, Jacobs DJ, Marquart L. Plausible mechanisms for the protectiveness of whole grains. The American Journal of Clinical Nutrition. 1999;70(3 suppl):459S - 63S.
8. Jenkins D, Kendall C, Axelsen M, Augustin L, Vuksan V. Viscous and nonviscous fibres, nonabsorbable and low glycaemic index carbohydrates, blood lipids and coronary heart disease. Current Opinion in Lipidology. 2000;11(1):49 - 56.

These two articles discuss possible physiological mechanisms in how can improve insulin sensitivity, and improve cholesterol profiles.

9. Kwiterovich PJ. The role of fiber in the treatment of hypercholesterolemia in children and adolescents. Pediatrics. 1995;96(5 Pt 2):1005 - 9.

This article shows the safe use of psyllium (a form of dietary fibre) in children to treat high cholesterol.

A2. Scientific background of the research

A2.1 Has this project been scientifically assessed by independent review? [X] Yes [ ] No

If **yes**, describe the process, for example, HRC funding assessment process. *A copy of the report should also be attached. The researcher's response may also be included.*	The research will be funded by the Australasian Paediatric Endocrine group following competitive application for a research grant to this regional society. It underwent scientific review as part of this process by multiple reviewers. Their reports are not provided to applicants.
If **no**, do you intend to have the project scientifically assessed and by whom?	

A2.2 Describe the scientific basis of the project **(300 words maximum)**. Where this space is inadequate, continue on a separate sheet of paper. *Do not* delete page breaks or renumber pages.

Recent literature has recognised that insulin resistance begins in childhood and extends into adulthood(1). Insulin resistance is a predictor for type 2 diabetes (T2DM) (2) and is often associated with obesity, particularly in adolescent onset type 2 diabetes in children (3-5). Furthermore insulin resistance alone is an independent risk factor for the development of the metabolic syndrome, hypertension, malignancy, stroke and heart disease (6). As cardiovascular disease remains the number one cause of death in New Zealand, research into prevention of T2DM is paramount(7).

Effective prevention of T2DM requires targeting of insulin resistance through community based healthy lifestyle interventions that include exercise, education and diet. Diet interventions can either consist of a hypocaloric diet, where insulin resistance is improved through weight loss, or macronutrient manipulation that could improve insulin resistance independently of weight loss. These two dietary strategies are not incompatible, and could be used in combination. Dietary macronutrient manipulation is likely to be more acceptable to adolescents, compared to caloric restriction in which compliance is universally poor. Nutritional management can vary greatly as there is a lack of consensus regarding what is the best approach (8). The results of this study will help clarify the role of fibre in nutritional management of obesity and insulin resistance.

In adults, *high-fibre* diets has been shown to improve S_I (9-12). However the 2008 Cochrane review could not determine if a *high-fibre* diet could prevent T2DM (13). In adolescents, increased whole grain diet as assessed by diet questionnaire showed improved S_I and BMI (9). This relationship was strongest in the obese patients, though this was not a randomised trial of fibre content and was based upon imprecise distant dietary recall.

At present there is no prospective data examining the effects of high fibre diet interventions alone on S_I, lipids and cardiovascular function such as blood pressure and arterial distensibility in adolescents.

1. Sinaiko A, Steinberger J, Moran A, Hong C, Prineas R, Jacobs DJ. Influence of insulin resistance and body mass index at age 13 on systolic blood pressure, triglycerides, and high-density lipoprotein cholesterol at age 19. Hypertension. 2006;48:730 - 6.
2. Mokdad O, Ford E, Bowman B, Dietz W, Vinicor F, Bales V, et al. Prevalence of obesity, diabetes, and obesity related health risk factors. Journal of the American Medical Association. 2001;289(1):76 - 9.
3. Steinberger J, Moran A, Hong C, Jacobs DJ, Sinaiko A. Adiposity in childhood predicts obesity and insulin resistance in young adulthood. Journal of Pediatrics. 2001;138(4):469 - 73.
4. Arslanian S, Suprasongsin C. Insulin sensitivity, lipids, and body composition in childhood: is "syndrome X" present? The Journal of Clinical Endocrinology and Metabolism. 1996;81(3):1058 - 62.
5. Caprio S, Bronson M, Sherwin R, Rife F, Tamborlane W. Co-existence of severe insulin resistance and hyperinsulinaemia in pre-adolescent obese children. Diabetologia. 1996;39(12):1489 - 97.
6. Saely C, Aczel S, Marte T, Langer P, Hoefle G, Drexel H. The metabolic syndrome, insulin resistance, and cardiovascular risk in diabetic and nondiabetic patients. The Journal of Clinical Endocrinology and Metabolism. 2005;90(10):5698 - 703.
7. New Zealand Ministry of Health. Ministry of Health Mortality Data. 2008.
8. Thomas D, Elliot E, Baur L. Low glycaemic index of low glycaemic load diets for overweight and obesity. Cochrane Database of Systematic Reviews. 2007(3):CD005105.
9. Steffen L, Jacobs D, Murtaugh M, Moran A, Steinberger J, Hong C, et al. Whole grain intake is associated with lower body mass and greater insulin sensitivity among adolescents. American Journal of Epidemiology. 2003;158(3):243 - 50.
10. McKeown N, Meigs J, Liu S, Wilson P, Jacques P. Whole-grain intake is favourably associated with metabolic risk factors for type 2 diabetes and cardiovascular disease in the Framingham offspring study. The American Journal of Clinical Nutrition. 2002;76(2):390 - 8.
11. Pereira M, Jacobs DJ, Pins J, Raatz S, Gross M, Slavin J, et al. Effect of whole grains on insulin sensitivity in overweight hyperinsulinemic adults. The American Journal of Clinical Nutrition. 2002;75(5):848 - 55.
12. Weickert M, Möhlig M, Schöfl C, Arafat A, Otto B, Viehoff H, et al. Cereal fiber improves whole-body insulin sensitivity in overweight and obese women. Diabetes Care. 2006;29(4):775 - 80.
13. Priebe M, van Binsbergen J, de Vos R, Vonk R. Whole grain foods for the prevention of type 2 diabetes mellitus. Cochrane Database of Systematic Reviews. 2008(1):CD006061.

A3. Study design

A3.1 Describe the study design. Where this space is inadequate, continue on a separate sheet of paper. *Do not* delete page breaks or renumber pages.

A 12-week blinded randomized cross-over trial of 6 weeks of either (a) a supplemented high fibre isocaloric diet compared to (b) an isocaloric diet alone in children and adolescents aged 13-19 years with obesity. After 6 weeks subjects in the supplemented high fibre diet will cross over to the normal diet and the normal diet subjects will cross over to the supplemented high fibre diet. Supplemental fibre will be in the form of 6g/day of psyllium, a naturally occurring form of fibre available over the counter and in health food shops. Psyllium is found in some breakfast cereals and high fibre breads and is derived from the husk of plantago seeds.

All subjects will be formally assessed at 3 time points; 1) baseline, 2) cross-over (at 6 weeks) and 3) study completion (12 weeks). Subjects will be randomised to receive either psyllium (6 gm/day) or no treatment for 6 weeks and then will be crossed over to the other treatment regimen for 6 weeks. There will be a two week washout period.

Each assessment will performed at the Liggins Institute (Maurice and Nessie Paykel Clinical Research Unit), and will include height measurement by wall-mounted stadiometer to the nearest 0.1cm, and weight measurement to nearest 0.1kg by electronic scale.

At each assessment subjects will be fasted overnight and have a 1.75gm/kg (max 75gm) glucodex oral glucose tolerance test (OGTT) performed with glucose and insulin levels (0, 30, 60, 90 and 120 min) determined and S_I measured by the Matsuda method. The Matsuda index of insulin sensitivity is a gold standard method to measure insulin sensitivity and is highly correlated with clamp derived insulin sensitivity indices. Other serum metabolic measures will be included including blood lipid profiles.

At each assessment arterial distensibility will be measured. This is facilitated by a blood pressure cuff and an external probe that measures the pulse wave form.

A DEXA body composition scan will occur at baseline, cross over, and at completion of the study (12 weeks).

Ambulatory blood pressure will be measured at baseline, cross over, and study completion.

A measured psyllium quantity is the most reliable way of delivering a precise dose of dietary fibre. We have chosen not to use a diet rich in psyllium containing foods (eg breakfast cereals) in the treatment group, as this would lead to a varying intake of psyllium and therefore difficulty in assessing the dose effect on insulin sensitivity. Each dose of psyllium will be measured and individually packaged and loaded into pill packs. The control (no psyllium) group will receive sugar-free, colour-free, drink sweetener similarly measured, packaged and loaded into pill packs. The use of pills packs will allow us to precisely monitor compliance with fibre intake.

Although no study has previously demonstrated significant side effects with high fibre diets, at each assessment markers of liver function, 25-OH vitamin D and ferritin and iron levels will be recorded.

Inclusion criteria

1. Age 13-19 years and without other significant medical illnesses.
2. Decile 1-3 high schools affiliated with the Liggins Institute Educational Network of Science (LENScience) such as Tamaki College. There are approximately 20 high schools affiliated with this programme of which 40% are low decile schools

Exclusion Criteria

1. Receiving medications that influence insulin sensitivity (including oral steroids and metformin).
2. Genetic conditions, (Turner, Downs or Prader-Willi syndrome)
3. Coeliac disease or other malabsorption disorders

A4. Participants

A4.1 How many participants do you intend to recruit? (Include details for each locality organisation.)

45 participants with justification provided in A4.2 below.

Students from decile 1-3 schools are likely to have high rates of obesity expressed as approximately 1/3 normal weight, 1/3 overweight and 1/3 obese. Thus recruitment of participants in such schools will provide significant representation of overweight and obese adolescents. The study is intended to determine whether there is improvement in insulin sensitivity in non-obese and obese children. Such a strategy will avoid the stigma of only recruiting obese adolescents.

A4.2 Give a justification for the number of research participants proposed, giving the details of power calculations when appropriate.

Based upon the Matsuda derived whole body insulin sensitivity index in a young adult population of 15.6 ±8.7, a 25% difference in S_I after periods with and without psyllium will require 41 subjects longitudinally studied with 80% power and a significance value of 0.05 (1). This assumes a correlation of 0.5 between measurements on the same person. We will enrol 45 subjects assuming a 10% drop out during the study.

1. Maki K, McKenney J, Farmer M, Reeves M, Dicklin M. Indices of insulin sensitivity and secretion from a standard liquid meal test in subjects with type 2 diabetes, impaired or normal fasting glucose. Nutrition Journal. 2009;8(22):1 - 10.

A4.3 If randomisation is used, explain how this will be done.

Randomization will utilize computer generated lists of random numbers.

A5. Statistical method

A5.1 Is the method of analysis quantitative? [X] Yes [ ] No

Or qualitative? [ ] Yes [ ] No

If the method of analysis is **wholly qualitative**, go to question A5.4.
If the method of analysis is **wholly or partly quantitative**, complete the following:

A5.2 Describe the statistical method that will be used to analyse the data.

Descriptive statistics such as means and percentages will be used to describe the baseline characteristics of the population General linear mixed models will be used to investigate the difference in SI at the end of the periods with and without psyllium . These models will enable us to model the covariance structure of the repeated measures on the subjects. Models will also include the period and baseline measurements as well as appropriate demographic measures . Similar models will be used to investigate differences in secondary outcomes such as body composition, arterial distensibility ,lipids and blood pressure.

A5.3 Has specialist statistical advice been obtained about this study? [X] Yes [ ] No

If **yes**, from whom? (A brief statistical report should be included if appropriate.)	Elizabeth Robinsons. Statistical report pending – available for the meeting.

A5.4 If the method of analysis is **wholly or partly qualitative**, specify the method. Why is this method appropriate? If interviews are to be used, include the general areas around which they will be based and a copy of the interview guide, if one is to be used. Copies of any questionnaires that will be used must be included.

A6. Expected outcomes or impacts of research

A6.1 What is the potential significance of this project for improved health outcomes?

Should a high fibre diet improve insulin sensitivity in obese children at risk of developing T2DM, and in healthy adolescents, then this would strengthen public health promotion across the community that a high fibre diet (eg cereals rich in psyllium) in children is an effective strategy in preventing insulin resistance and its long term sequelae.

A6.2 What is the potential significance of this project for the advancement of knowledge?

At present there is a paucity of quality data examining the influence of dietary changes at a macronutrient level on insulin sensitivity and obesity in children and adolescents. As such there is a lack of consensus regarding the dietary management of obesity in this population. This study will help to clarify whether a high fibre diet, independent of any other intervention, can improve insulin sensitivity and/or BMI.

A6.3 What steps will be taken to disseminate the research results?

Publish in scientific journals.

Present results at local and international conferences.

A7. Publication of results

Will any restriction be placed on publication of results? [ ] Yes [X] No

If **yes**, please supply details.

A8. Funding

A8.1 How will the project be funded?

Laboratory costs:

The Clinical Research Centre costs are met by the Liggins Institute and will include room and equipment access and the assistance of a research nurse for recruitment and growth measurements. Grant applications are in process to aid in funding.

An Australasian Paediatric Endocrine Society grant of AUS$25,000 has been awarded which will be used for research costs.

A NZSSD grant of $10,000 has been awarded which will be used for research costs.

Salary for researcher: Liggins Institute. Ongoing applications for research grants.

A8.2 Does the researcher, the host department, the host institution or the locality organisation have any conflict of interest, eg, financial interest, in the outcome of this research? If **yes**, please give details. [ ] Yes [X] No

A9. Incentive payments

A9.1 Have you read and understood the description of incentive payments in the Guidelines? [X] Yes [ ] No

Note: Details about any payment (in money or kind) or reward made to participants recruited into the project are to be provided in question E10.

A9.2 Does the funding available to the project depend upon the number of participants [ ] Yes [X] No

recruited, eg, is the funding on a per participant basis?. If **yes**, give details of the amount per participant. Where there is a significant difference between these, this incentive to recruit should be declared in the information sheet.

A9.3 Does the funding available to the project include any form of incentive (in money or kind) for the early or complete recruitment of a specified number of participants, eg, bonus payments to the researcher, host department or host institution? If **yes**, give details.

[ ] Yes [X] No

A9.4 Will **all** funding available to the project be passed through an audited research account or cost centre? If **yes**, give details. If **no**, specify why not.

[X] Yes [ ] No

The research project grant account will be a University of Auckland Research Office grant account.

B. Minimisation of harm *(Operational standard paragraphs 60–68)*

B1. How many visits/admissions of participants will this study involve? Clarify what is in addition to standard treatment. Give also an estimate of total time involved for participants.

Each participant will have 3 visits to the Liggins. They participant will have to come in fasted. An oral glucose tolerance test will be administered as outlined above. Participants will be advised that each visit will take 3 hours. These visits are additional to standard treatment for obesity (if the participant is actually obese). Therefore total time involved for particpants is estimated to be 10 hours.

B2. Who will carry out the research procedures?

Dr Martin de Bock

B3. What other research studies is the lead investigator currently involved with?

None

B4. Where will the research procedures take place?

Liggins Institute.

B5. How do the research procedures differ from standard treatment procedures?

Standard treatment of obesity does not involve repeated formal testing for insulin sensitivity, making the three visits additional. Many obese adolescents would have an oral glucose tolerance test performed as part of routine care by GP or specialist.

B6. What are the benefits to research participants of taking part in the project?

If fibre improves insulin sensitivity then participants will have received some direct health benefit from participating in the study and at study completion will be given standard dietary and lifestyle advice with detailed information about the possible benefit of a high fibre diet for them based upon their study results.<br><br>Insulin sensitivity testing does involve IV access with a single iv used for all blood sampling and blood sampling after an oral glucose tolerance test. This intervention carries minimal risk, and only mild discomfort.

B7. Describe any methods for obtaining information. Attach questionnaires and interview guidelines. (If National Health Index (NHI) information is used, see the Guidelines (NAFG-2009-v1).)

NHI data will be used for laboratory assays. This will be unlinked. Due to randomization, participants will be given a number, which is the what all data will be recorded against. No records of names, or addresses will be linked with the data collected. Data will be stored on a secured computer network which is password protected at the Liggins Institute.<br><br>The study population has a high proportion of Maori and Pacific. Ethnicity will be recorded. However, as ethnic groups, privacy will be protected in the same way in which individual data is protected.

B8. Briefly describe the inclusion/exclusion criteria and include the relevant page number(s) of the protocol or investigator's brochure.

Inclusion criteria

1. Age 13-19years and without other significant medical illnesses.
2. Attend Decile 1-3 high schools affiliated with the Liggins Institute Educational Network of Science (LENScience) such as Tamaki College. There are approximately 20 high schools affiliated with this programme of which 40% are low decile schools

Exclusion Criteria

1. Receiving medications that influence S_I (incluing oral steroids and metformin).
2. Genetic conditions, (Turner, Downs or Prader-Willi syndrome)
3. Coeliac disease or other malabsorption disorders

B9. What are the physical or psychological risks or side effects to participants or third parties? Describe what action will be taken to minimise any such risks or side effects.

Physical risks:

There have been no reported adverse affects documented by previous authors using equal doses of dietary fibre in children and adolescents. An increase in stool volume, frequency and flatus can be expected, and this will be discussed with potential participants during recruitment and consent.

The oral glucose tolerance test and insulin sensitivity assay has a small risk around IV access. The researcher has 7 years experience in clinical medicine, where IV access is a well developed skill, which will minimise the risk of failed IV insertion. The risk of tissue damage is negligible.

Psychological:

Because the study has an obesity aspect, there is potential that participants may develop anxiety about their weight or body image during the study. As this is a school based population, the researcher will develop a relationship with the school guidance counsellor, and alert them to the study. The researcher has experience in adolescent mental health, and is equipped to identify any issues, and could facilitate engagement with the school guidance counsellor if indicated.

B10. What facilities/procedures and personnel are there for dealing with emergencies?

The Liggins Institute has research nurses available for hands on help.

The investigation unit has first aid facilities.

Auckland Hospital is based 500m from the Liggins Institute

The researcher is an advanced trainee in paediatrics, who has 7 years of experience in medicine, including significant training in emergency care. The principal researcher has a current Advanced Paediatric Life Support certification.

B11. What arrangements will be made for monitoring and detecting adverse outcomes?

There have been no adverse outcomes described in the literature for children and adolescents supplemented with dietary fibre.

Nevertheless at each assessment markers of liver function, 25-OH vitamin D and ferritin and iron levels will be recorded. Any significant changes in levels will be reported.

B12. If the study is a clinical trial, are participants to be provided with a card confirming their participation, medication and the contact phone number of the principal investigator? [X] Yes [ ] No

B12.1 Do you intend to inform the participant's GP that their patient is a participant in this study? (If yes, consent from the participant is required.) [ ] Yes [X] No

B12.2 Do you intend to inform the GP of all clinically significant abnormal results obtained during study conduct? [X] Yes [ ] No

B13. Is the trial being reviewed by a data and safety monitoring board (DSMB)? ☐ Yes ☒ No

If **yes**, who is the funder of the DSMB? ☐ HRC ☐ Sponsor ☐ Other

If **'Other'**, please specify.

B14. What are the criteria for terminating the study?

1. Essentially there are none as the study involves administration of a healthy food product (dietary fibre).
2. Inability to recruit participants (extremely unlikely)
3. Study participants increasing absence from school
4. Unexpected adverse laboratory monitoring data (of liver function, 25-OH vitamin D and ferritin and iron levels), not previously reported with high fibre diets.

B15. Will participants be exposed to any potential toxins, mutagens or teratogens? ☐ Yes ☒ No

If **yes**, specify and outline the justification for their use.

B16. Will any radiation or radioactive substances be used? ☒ Yes ☐ No
Note: If any form of radiation is being used, please answer B16.1–B16.2.
If no, go to question B17.

B16.1 How many x-rays or other procedures are planned for the purposes of this study, ie, that are not part of standard treatment?

Three DEXA scans (at baseline, at 6 weeks and at study completion at 12 weeks)

B16.2 Under whose licence is the radiation being used?

Paul Hofman

B16.3 Has the National Radiation Laboratory (NRL) risk assessment been completed? ☐ Yes ☒ No

If **yes**, please enclose a copy of the risk assessment and a contact name and phone number.

If **no**, please explain why not.

DEXA scans have extremely low fixed radiation exposure. The dose is similar to that obtained on an international flight and there is international consensus that they do not constitute a safety concern to children.

B17. Will any medicines be administered for the purposes of this study? ☐ Yes ☒ No

An over the counter formulation of psyllium will be given

B17.1 If **yes,** is Standing Committee on Therapeutic Trials (SCOTT) approval required? ☐ Yes ☒ No

B17.2 Has SCOTT approval been given? (Please attach.) ☐ Yes ☐ No

B18. Does the study involve the use of health care resources? ☐ Yes ☒ No
If **yes**, please specify:

B19. What effect will this use of resources have on waiting list times for patients, that is, for diagnostic tests or for standard treatments?

No effect

C. Compensation for harm suffered by participants

(Operational standard paragraphs 87–95)

(Refer also to Appendix 3 of the Guidelines (NAFG-2009-v1).)

C1. Will participants be treated by, or at the direction of, a registered health professional as part of the research? (Treatment includes screening, diagnosis, for definitions see the Guidelines (NAFG-2009-v1) pages 11-13.) [X] Yes [ ] No

If **no**, go to section D. If **yes**, please answer questions C2–C6.4.

C2. Is the research being carried out principally for the benefit of a manufacturer or distributor of the drug or item in respect of which the research is taking place? [ ] Yes [X] No

C2.1 If the answer to C2 is **yes**, please complete **Statutory Declaration Form B** and answer questions C3–C6.4.

C2.2 If the answer to C2 is **no,** please complete **Statutory Declaration Form A** and go to section D.

Depending on all the circumstances, the minimum cover that is likely to be acceptable to the ethics committee is that provided under ACC. In any case, all exclusions to compensation must be clearly and explicitly set out in the participant information sheet, including those that may be described in C5.

C3. Is the manufacturer/distributor's agreement to provide compensation in accordance with the RMI attached? [ ] Yes [ ] No

C4. Has the manufacturer or distributor agreed to cover any injury/adverse consequence resulting from participation in this research? [ ] Yes [ ] No

C4.1 If no, what qualifications have been specified for cover?

C4.2 **Limiting the type of compensation**

C4.2.1 Has the manufacturer or distributor excluded any type of compensation, for example, pain and suffering, loss of earnings, loss of earning capacity, funeral costs, dependents' allowances or any other financial loss or expenses? ☐ Yes ☐ No

C4.2.2 If yes, please state what is excluded. (Include in the compensation statement on the information sheet)

C5. **Limiting liability – exclusion clauses**

C5.1 Has the manufacturer or distributor limited or excluded liability if the injury is attributable to the negligence of someone other than the manufacturer or distributor (such as negligence by the investigator, research staff, the hospital or institution, or the participant)? ☐ Yes ☐ No

C5.2 Has the manufacturer or distributor limited or excluded liability if the if the injury resulted from a significant deviation from the study protocol by someone other than the manufacturer or distributor? ☐ Yes ☐ No

C5.3 Is evidence of the following indemnity insurance attached?

Sponsor ☐ Yes ☐ No

If yes to either C5.1 or C5.2;

Hospital/institution ☐ Yes ☐ No

Investigator ☐ Yes ☐ No

C5.4 Is company liability limited in any other way? ☐ Yes ☐ No

If **yes**, please specify.

D. Privacy and confidentiality *(Operational standard paragraphs 48–56)*

D1. How will potential participants be identified?	Patients will be recruited from Decile 1-3 high schools affiliated with the Liggins Institute Educational Network of Science (LENScience) such as Tamaki College. There are approximately 20 high schools affiliated with this programme of which 40% are low decile schools, which have high rates of obesity. As there are no other inclusion criteria required and the entire roll will be potential participants
D2. How will participants be recruited (for example, advertisements, notices)?	There is a working relationship between the school and the Liggins Institute. Advertising will be through school notices, and face to face recruitment by addressing classes. It is expected that this study could link in with school curriculum on healthy lifestyle, and therefore these classes would be identified as best recruiting oppurtunities.
D3. Where will potential participants be approached (for example, outpatient clinic)? If appropriate, describe by type (for example, students).	Decile 1-3 high schools affiliated with the Liggins Institute Educational Network of Science (LENScience) such as Tamaki College. There are approximately 20 high schools affiliated with this programme of which 40% are low decile schools. The programme is run by Mrs Jacquie bay an advisor to the study.<br>It is conceivable that project participation may become a component of the LENScience experiential programme of engaging in biomedical research
D4. Who will make the initial approach to potential participants?	The College principal through their biology teaching staff.

NB: Do not include information on storage and use of tissue samples and related information in the following questions. That is covered separately under Part 5.

D5. How will data, including audio- and videotapes, be handled and stored to safeguard confidentiality (both during and after completion of the research project)?	Data will be collected and stored electronically and a secure password protected network at the Liggins Institute. All data will be de-personalised, with participants being assigned a number for identification.
D6. What will be done with the raw data when the study is finished?	Once statistical analysis is finished, the raw data will be stored on a secure electronic server at the Liggins Insititute.
D7. How long will the data from the study be kept, and who will be responsible for their safe keeping? (Health information relating to an identifiable individual must be retained for at least 10 years, or in the case of a child, 10 years from the age of 16.)	Data will be kept after completion of the study for 10 years.

D8.	Name those who will have access to the raw data, participant information and/or clinical records during, or after, the study?	Dr Martin de Bock<br>Prof Wayne Cutfield<br>Dr Paul Hofman<br>Mrs Elizabeth Robinson
D9.	Describe any arrangements to make results available to participants, including whether they will be offered their audio- or videotapes.	After finalising the data, I will present the results to those who are interested from the participating schools.

E. Informed consent *(Operational standard paragraphs 28–43)*

A participant's informed consent should be obtained in writing, unless the procedures are not experimental and there are good reasons for not requiring written consent. If consent is not to be obtained in writing, the justification should be given and the circumstances under which consent is obtained should be recorded. Attach a copy of the information sheet and consent form provided to participants.

E1.	By whom, and how, will the project be explained to potential participants?	Dr Martin de Bock will explain the project.<br>The study will be presented verbally using non medical language. This will be followed up with an information leaflet, also using plain language, for potential participants to read and to take home.
E2.	When and where will the explanation be given?	Explanation will occur at the participating school, in the classroom, to fit in with current curriculum on health.

E3. Will a competent interpreter be available, if required? [X] Yes [ ] No

If **no**, why not?

E4.	How much time will be allowed for the potential participant to decide about taking part in the project?	One week.
E5.	In what form (written, or oral) will consent be obtained? If oral consent only, state reasons.	Written consent

E6. If recordings are made, will participants be offered the opportunity to edit the transcripts of the recordings? [X] N/A [ ] No

E7. Will data or other information be stored for use in a different study for which ethics committee approval would be required? [ ] Yes [x] No

E7.1 If **yes**, please explain how.	
E8. Is there any special relationship between the participants and the researchers (for example, doctor/patient, student/teacher)?	No
E9. Will there be any financial cost to the participant, for example, travel and parking costs? If so, will such cost be reimbursed? (Refer to the Guidelines (NAFG-2009-v1).)	Yes. Cost for parking, travel and breakfast for this fasting study will be reimbursed.

E10. Will any payments be made to participants, or will they gain materially in other ways from participating in this project? [ ] Yes [X] No

E10.1 If **yes**, please supply details.	

F. Cultural and social responsibility *(Operational standard paragraphs 73–82)*

Section F enshrines two fundamental principles. They are:

i. Culturally safe research practice: Research involving participants from specific ethnic or socially identified groups (even when small numbers from each group are involved) must involve those participant groups in the research process as full participants. Where a particular ethnic or socially identified group is the principal subject of the research, there must be engagement with appropriate parties, and this process must be outlined in the application.

ii. If the research is in an area of health inequalities, then the researcher must demonstrate how the research will contribute to achieving equity of outcomes for those population groups most in need within the public good health system.

F1. Have you read the HRC booklet *Guidelines for Researchers on Health Research Involving Māori*? [X] Yes [ ] No

Relevance and responsiveness to Māori

F2. **All health research conducted in Aotearoa New Zealand is of relevance to Māori**. How relevant is a decision to be made by Māori. The researcher must be able to articulate the context and the relevance of the proposed research to Māori and the possible consequences for Māori health outcomes, and generally, the greater the degree of relevance to Māori, the greater the expectation of participation of Māori and hence consultation expectations.

F2.1 Given your approach to sampling, what are the anticipated numbers of Māori participants?	15 out of 40
F2.2 What is the incidence among Māori of the health issue/disability relevant to the study?	35% of Maori school children are overweight.<br><br>Tyrell V, Richards G, Hofman P, Gillies G, Robinson E, Cutfield W. Obesity in Auckland school children: a comparison of the body mass index and percentage body fat as the diagnostic criterion. International Journal of Obesity. 2001;25:164 - 9.

F3. Please explain how this research will contribute to improving Māori health outcomes and reducing health inequalities for Māori.

> This study will involve a high proportion of Māori compared to the population as a whole. The significance of this study is to look at ways in which insulin sensitivity can be improved – a problem often associated with obesity. Obesity occurs at a higher rate amongst Māori as does type II diabetes. The ultimate endpoint of diabetes is cardiovascular and renal disease which is disproportionately represented in the Māori population, and remains the number one cause of death in New Zealand.
>
> Therefore, finding ways to improve insulin sensitivity will directly improve Māori health by improving rates of obesity, type II diabetes, and cardiovascular disease – all of which are examples of health inequalities for Māori.

F4. Describe the process by which Māori have been engaged in the conception and design of the proposed research. Please identify the group/s with which consultation has taken place and outline their stated view about the proposed research. Please attach their letter/s of support for this specific research project.

> I have consulted with Mr Jim Peter, the Maori ProVice-Chancellor at the University of Auckland. A letter of support for the research has been received and is attached.
>
> Some Iwi disagree with the storage of tissue or blood samples citing whakapapa, and advise their people to consult prior to participation in research where this occurs. However, it is acknowledged that individuals have the right to choose to participate.

F4.1 Describe any ongoing involvement the group(s) consulted have in the project.

> The support letter allows for further consultation if required.

F4.2 Describe how information will be disseminated to participants and the group(s) consulted during and at the conclusion of the research project.

> Participants will be invited to attend a presentation of the data at participating Colleges. This opportunity will be open to consulted groups.

Responsiveness to ethnic peoples

F5. What other ethnic groups will be participating in this research based on your sampling frame (for example, Pacific peoples or Asian peoples)?

> Pacific
>
> Asian

F5.1 Are there any aspects of the research based on participation or the relevance of the research to specific ethnic groups that might raise specific cultural issues?

[X] Yes [ ] No

If **yes**, please outline.
If **no**, go to F6.

It is already established that the population that is being recruited has a high proportion Māori and Pacific people, and a high proportion of obesity. Healthy lifestyle including exercise and diet is already identified as a cultural issue, and this study will address these issues indirectly.

F5.2 How can this research contribute to reducing inequalities for ethnic peoples in the New Zealand health system?

Because there is a high proportion of obesity, and therefore insulin resistance amount the ethnic people in New Zealand, interventions to improve this will directly improve inequalities in obesity, insulin resistance, diabetes, and cardiovascular disease.

F5.3 Describe what consultation has taken place with specific ethnic group(s) prior to the project's development and attach evidence of their support.

F5.4 Describe any ongoing involvement the group(s) consulted have in the project.

F5.5 Describe how you intend to disseminate information to participants and the group(s) consulted at the end of the project.

Participants will be invited to attend a presentation of the data at participating Colleges. This opportunity will be open to consulted groups.

Responsiveness to other peoples of interest

F6. Are there any aspects of the research based on participation or the relevance of the research to specific peoples of interest that might raise specific issues for such communities (for example, for prisoners, people with disabilities, people with diverse sexual identities)?

[ ] Yes [X] No

If **yes**, please outline.
If **no**, go to F7.

F6.1 How can this research contribute to reducing inequalities for other peoples of interest in the New Zealand health system?

F6.2 Describe what consultation has taken place with specific peoples of interest group(s) prior to the project's development and attach evidence of their support.

F6.3 Describe any ongoing involvement the group(s) consulted have in the project.

F6.4 Describe how you intend to disseminate information to participants and the group(s) consulted at the end of the project.

F7. Will the study drug/treatment continue to be available to the participant after the study ends? [X] Yes [ ] No

F7.1 If **yes**, will there be a cost, and how will this be met?	This will be to the cost of the participant. Cost to patient is $9.80 for 25 doses when purchased over the counter from health food shops and chemists.
F7.2 If **no**, why not?	
F7.3 If there was a placebo arm, what will happen to these participants at the end of the study?	As this is a cross over design all subjects will benefit from a high fibre diet for half the study.

Note: This information needs to be included in the information sheet.

Part 3: General

Describe and discuss any ethical issues arising from this project, other than those already dealt with in your answers above.

Thank you for your assistance in helping us assess your project fully.

Please now complete:

- the declarations (Part 4). If there is more than one site, include a declaration for each site.

If applicable complete:

- a Registered Drug Form
- Form A or B
- Part 5
- Part 6
- Part 7
- Part 8

Attach:

- Checklist to ensure all relevant documents are attached. Incomplete applications will not be reviewed.

Part 4: Declarations

Full project title:	**Can supplemented dietary fibre improve insulin sensitivity in obese adolescents?**<br>*A randomized cross-over trial of a high fibre isocaloric diet compared to an isocaloric diet alone in a secondary school community with a high incidence of obesity*
Short project title:	The impact of supplemented fibre on insulin sensitivity in adolescents.

1. Declaration by principal investigator

The information supplied in this application is, to the best of my knowledge and belief, accurate. I have considered the ethical issues involved in this research and believe that I have adequately addressed them in this application. I understand that if the protocol for this research changes in any way, I must inform the ethics committee.

Name of Principal Investigator (please print):	Martin de Bock
Signature of Principal Investigator:	
Date:	

2. Declaration by Head of Department in which the Principal Investigator is located or appropriate Dean or other Senior Manager

I have read the application, and it is appropriate for this research to be conducted in this department. I give my consent for the application to be forwarded to the ethics committee.

Name (please print):	Prof Murray Mitchell		
Signature:		Institution:	Liggins Institute
Date:	20/11/09	Designation:	Deputy Director

- Where the Head of Department is also one of the investigators, the Head of Department declaration must be signed by the appropriate Dean, or other senior manager.
- If the application is for a student project, the supervisor should sign the Head of Department declaration.
- Submit a declaration by the principal investigator for each site.

3. Locality organisation approval

Locality organisation approval is being attached from the following locations: Liggins Institute

Form A: Declaration of Eligibility of a Clinical Trial for Consideration of Coverage under Accident Compensation Legislation

Instructions: This form is to be completed and the statutory declaration signed by the most senior registered health professional providing or directing the provision of treatment as part of the research. It should be forwarded to the appropriate ethics committee together with the documents seeking ethical approval for the proposed study. The information provided must be sufficiently detailed to enable the ethics committee to be satisfied that the proposed research is not conducted principally for the benefit of the manufacturer or distributor of the medicine or item in respect of which the research is carried out.

The provision of this information will enable the ethics committee to be satisfied that participants in the clinical trial will be considered for coverage under accident compensation legislation for injury caused as a result of their participation in the research.

Details of proposed research study

Title of research project:	**Can supplemented dietary fibre improve insulin sensitivity in obese adolescents?**<br><br>*A randomized cross-over trial of a high fibre isocaloric diet compared to an isocaloric diet alone in a secondary school community with a high incidence of obesity*
Name of research director/investigator:	Dr Martin de Bock
Location/s of proposed study:	Liggins Institute, Auckland
Number of participants:	45
Organisations providing support (in money or kind) for the direct and indirect costs of the research *(please provide names of organisations and details of the type of support provided)*:	Liggins Institute, Auckland
Relationship of proposed research to the pharmaceutical industry or other company involved in health research *(please describe the involvement of industry in your proposed research and provide details of support to be received from them)*:	There is no relationship with the pharmaceutical industry. Psyllium a form of dietary fibre will be independently dispensed.

Statutory declaration

I Martin de Bock of Auckland solemnly and sincerely declare that as the most senior registered health professional providing or directing the provision of treatment as part of the research, the proposed study is not conducted principally for the benefit of the manufacturer or distributor of the medicine or item in respect of which the trial is carried out. I make this solemn declaration conscientiously believing the same to be true and by virtue of the Oaths and Declarations Act 1957.

Name *Martin de Bock* | Signature | this day of

before me

Name of witness *(please print)* | Signature of witness

a Justice of the Peace, or ☐

a Solicitor of the High Court ☐

or other person authorised to take a statutory declaration. ☐

Warning: Please note that it is an offence under part VI subsection 111 of the Crimes Act 1961 to make a false statutory declaration. **Note:** Applicants conducting a research study that is conducted principally for the benefit of the manufacturer or distributor of the medicine or item in respect of which the trial is carried out should complete Form B.
